# Sensory and instrumental analysis of medium and long shelf-life Charentais cantaloupe melons (*Cucumis melo* L.) harvested at different maturities

**DOI:** 10.1016/j.foodchem.2013.10.045

**Published:** 2014-04-01

**Authors:** Stella Lignou, Jane K. Parker, Charles Baxter, Donald S. Mottram

**Affiliations:** a University of Reading, Department of Food and Nutritional Sciences, Whiteknights, Reading RG6 6AP, UK; b Syngenta Seeds Limited, Jealott’s Hill International Research Centre, Bracknell, Berkshire RG42 6EY, UK

**Keywords:** Melon, *Cucumis melo* L., Flavour, Cantaloupe, Charentais, Volatile compounds, Semi-volatile compounds, Sensory evaluation, GC–MS, GC–O/MS

## Abstract

The flavour profiles of two genotypes of Charentais cantaloupe melons (medium shelf-life and long shelf-life), harvested at two distinct maturities (immature and mature fruit), were investigated. Dynamic headspace extraction (DHE), solid-phase extraction (SPE), gas chromatography–mass spectrometry (GC–MS) and gas chromatography–olfactometry/mass spectrometry (GC–O/MS) were used to determine volatile and semi-volatile compounds. Qualitative descriptive analysis (QDA) was used to assess the organoleptic impact of the different melons and the sensory data were correlated with the chemical analysis. There were significant, consistent and substantial differences between the mature and immature fruit for the medium shelf-life genotype, the less mature giving a green, cucumber character and lacking the sweet, fruity character of the mature fruit. However, maturity at harvest had a much smaller impact on the long shelf-life melons and fewer differences were detected. These long shelf-life melons tasted sweet, but lacked fruity flavours, instead exhibiting a musty, earthy character.

## Highlights

• Flavour of medium and long shelf-life Charentais cantaloupe melons was compared.
• Volatile and semi-volatile profiles were correlated with sensory data using multifactorial analysis.
• Maturity at harvest has a significant impact on the flavour of medium-shelf life fruit.
• Maturity at harvest had much less impact on a long shelf-life genotype.
• Esters and sulphur-compounds were more abundant in mature medium shelf-life fruit.

## 1 Introduction

Fully ripe orange-fleshed Charentais melons (*Cucumis melo* L. var. *cantalupensis*) are highly considered for their unique aromatic flavour as well as for the sweet taste of the flesh, both characteristics which develop as the fruit reaches full maturity. Volatile compounds, mainly esters, increase with increasing fruit maturity, thus contributing to the desirable sweet aroma of the fruit. Moreover, fruit that remains attached to the plant accumulates sucrose, resulting in a fruit with a sweet taste. Therefore, to achieve optimum quality and consumer acceptance, melon fruit should be harvested fully mature. Unfortunately, the shelf-life of Charentais melons tends to be very short. In order to deliver a longer shelf-life, fruits are either harvested partially mature, or varieties with extended shelf-life are used. Hybrids of the latter have been produced by plant breeders in order to extend the shelf-life, although consumers often complain about their poor quality, which is associated with less aroma, compared with wild-varieties (Aubert & Bourger, 2004).

There have been many studies investigating different types of melons, focusing on the effect of harvest maturity on quality characteristics, including colour, firmness, ethylene, total sugars, organic acids, amino acids, volatile compounds and sensory characteristics (Beaulieu, 2006; Beaulieu & Grimm, 2001; Beaulieu, Ingram, Lea, & Bett-Garber, 2004; Beaulieu & Lancaster, 2007; Beaulieu & Lea, 2007; Wang, Wyllie, & Leach, 1996; Wyllie, Leach, & Wang, 1996; Vallone, et al., 2013), but very few on Charentais melons (Alsmeirat & El-Assi, 2010; El-Assi & Alsmeirat, 2010). Moreover, there are several studies showing how volatile compounds decrease in Véndrantais melons transformed with an aminocyclopropane-1-carboxylic acid (ACC) oxidase antisense gene (Bauchot, Mottram, Dodson, & John, 1998; Bauchot, Mottram, & John, 2000), however, only a few papers focus on the volatile compounds of medium and long shelf-life varieties obtained by conventional breeding methods (Aubert & Bourger, 2004; Lamikanra, Juaraez, Watson, & Richard, 2003).

The purpose of this study was to investigate the effect of harvest maturity and the effect of two different genotypes of Charentais melons with extended shelf-life, on the flavour profile (volatile, semi-volatile and non-volatile compounds) of the melons. Moreover, quantitative descriptive analysis was also used in order to confirm the organoleptic impact of the chemical changes and to find correlations between sensory and instrumental data.

## 2 Materials and methods

### 2.1 Melons

Charentais melons (*C. melo* L. var. *cantalupensis*) of two different genotypes (one medium shelf-life coded as MSL (cv. Match) and one long shelf-life coded as LSL (cv. Vulcano)) harvested at two distinct maturities (immature – harvested prior to commercial harvest point – coded as i, and mature – harvested at commercial harvest point – coded as m) were supplied by Syngenta Seeds Ltd. The harvest point was defined according to the senescence of the leaf next to the fruit, also taking into account changes in the external fruit colour plus the senescence of the peduncle (these are non-slip varieties which means that they do not detach from the plant; however, the peduncle does senesce). Melons were stored at 8 °C before analysis, and all analyses were performed within 4 days of receipt in June 2009 (shipping times were the same for all samples and aligned to commercial practices).

### 2.2 Chemicals

For capillary electrophoresis (CE), the basic anion buffer (Part No.: 5064-8209) used for sugar and organic acid analysis was purchased from Agilent (Santa Clara, CA). Glucose, fructose, and citric acid were purchased from Sigma–Aldrich Co. Ltd. and sucrose and malic acid from Fluka (Poole, UK). For solid-phase extraction (SPE), HPLC-grade methanol was purchased from Merck Ltd. (Poole, UK) and methyl acetate, sodium sulphate and HPLC grade water from Fisher Scientific (Loughborough, UK). 3-Chlorophenol and the alkane standard C_7_–C_30_ (1000 μg/ml) in hexane were purchased from Sigma–Aldrich Co. Ltd. (Gillingham, UK). For dynamic headspace extraction (DHE), compounds used as standards were obtained from Sigma–Aldrich Co. Ltd. 1,2-dichlorobenzene in methanol (130.6 μg/ml) and the alkane standards C_6_–C_25_ (100 μg/ml) in diethyl ether. The EZ-Faast amino acid analysis kit (Phenomenex, Torrance, CA) was used for the analysis of amino acids by GC–MS. Norvaline was obtained from Sigma–Aldrich Co. Ltd.

### 2.3 Preparation of sample extracts

One melon from each point (maturity, genotype) was rinsed in cold running tap water, the skin (0.8 cm) and the seeds were removed and the remaining fruit was chopped and blended in a food processor. Portions of 200 g were weighed into polypropylene centrifuge bottles (250 ml; Nalge Nunc International, Rochester, NY) and the bottles were centrifuged at 21,859*g* for 20 min at 4 °C in a RC-6C Plus Sorvall R centrifuge (Thermo Scientific, Waltham, MA). For chemical analysis, the supernatant juice was filtered under vacuum using a Whatman filter No. 1 (GE Healthcare UK Ltd., Buckinghamshire, UK), in order to remove any tissue particles, and the filtrate was used for all the analyses. Three replicate fruits were prepared for each point. Portions of the 12 melon extracts were used immediately for sensory and volatile analysis, while the remainder was stored at −20 °C prior to semi-volatile and non-volatile analyses.

### 2.4 Volatile compounds

#### 2.4.1 Dynamic headspace extraction

Melon juice (2 ml) obtained as described above, was transferred to a 250-ml conical flask with a screw-thread neck and 10 ml of water were added. The flask was then placed in the water bath at 37 °C, and a flow of nitrogen swept the volatiles for 1 h at 40 ml/min onto a glass-lined, stainless steel trap (105 × 3 mm i.d.) containing 85 mg of Tenax TA (Scientific Glass Engineering Ltd, Ringwood, Australia). Internal standard (1 μl of 130.6 μg/ml 1,2-dichlorobenzene in methanol) was added to the trap at the end of the collection, and excess solvent and any water retained on the trap were removed by purging the trap with nitrogen at 100 ml/min for 10 min.

#### 2.4.2 GC–MS analysis of DHE extracts

Traps were thermally desorbed in a CHIS injection port (Scientific Glass Engineering Ltd) attached to a HP5890/5972 GC–MS (Agilent) as described by Elmore, Parker, Halford, Muttucumaru, and Mottram (2008). Volatiles were identified by comparison of each mass spectrum with spectra from authentic compounds analysed in our laboratory, or from the NIST mass spectral database (NIST/EPA/NIH Mass Spectral database, 2008), or spectra published elsewhere. To confirm the identification, the linear retention index (LRI) was calculated for each volatile compound using the retention times of a homologous series of C_6_–C_25_ *n*-alkanes and by comparing the LRI with those of authentic compounds analysed under similar conditions. The approximate quantification of volatiles collected from the headspace were calculated from GC peak areas, by comparison with the peak area of the 1,2-dichlorobenzene standard, using a response factor of 1.

#### 2.4.3 GC–O/MS analysis of DHE extracts

After the extraction onto preconditioned glass traps (4 mm i.d., 6 mm o.d., and 89 mm long) packed with Tenax TA (Supelco, Bellefonte, PA) as described above (but from 20 ml of melon juice), the trap was desorbed onto a HP-5MS column (30 m × 0.25 mm × 0.25 μm film thickness) in an Agilent 7890A/5975C GC–MS (Agilent, Santa Clara, CA), equipped with an automated thermal desorber (Turbomatrix ATD; Perkin Elmer, Waltham, MA) and fitted with an ODO 2 GC–O system (Scientific Glass Engineering Ltd.). After desorption, the oven was maintained at 40 °C for a further 2 min and then the temperature was raised at 4 °C/min to 300 °C. The mass spectrometer was operated in the electron impact mode with a source temperature of 230 °C, an ionising voltage of 70 eV, and a scan range from *m/z* 20 to 400. Two assessors were used for the detection and verbal description of the odour-active components of extracts and only those odours which were detected by both assessors were recorded in the results. The assessors scored each odour on a seven-point line-scale (2–8) where 3 = weak, 5 = medium and 7 = strong. *n*-Alkanes C_6_–C_25_ were analysed under the same conditions to obtain linear retention index (LRI) values for the components.

### 2.5 Semi-volatile compounds

#### 2.5.1 Solid-phase extraction

3-Chlorophenol (100 μl of a solution containing 1 mg/ml in 10% methanol/water) was added to the filtrate (20 ± 0.1 ml) as internal standard and the extraction was performed as described by Lignou, Parker, Oruna-Concha, and Mottram (2013).

#### 2.5.2 GC–MS analysis of SPE extracts

Extracts were analysed by an Agilent 6890/5975 GC–MS as described by Lignou et al. (2013). Semi-volatile compounds were identified as described above for the volatile compounds. The semi-quantification of semi-volatile compounds was calculated from the GC peak areas, by comparing with the peak area of the 3-chlorophenol standard, using a response factor of 1.

#### 2.5.3 GC–O/MS analysis of SPE extracts

The extract (1 μL) was injected into the injection port of an Agilent 7890A/5975C Series GC–MS system equipped with an ODO 2 GC–O system. The column used was a DB-Wax column (30 m × 0.25 mm × 0.25 μm film thickness). The temperature programme employed was 1 min at 40 °C, a ramp of 4 °C/min to 240 °C, and hold for 10 min. The extract was injected in splitless mode. The helium carrier gas flow rate was 1 ml/min. The mass spectrometer was operated in electron impact mode with a source temperature of 230 °C, an ionising voltage of 70 eV, and a scan range from *m/z* 29 to 400. One assessor was used for the detection and verbal description of the odour-active components of extracts. Each odour was scored on a seven-point line-scale (2–8) where 3 = weak, 5 = medium and 7 = strong. *n*-Alkanes C_7_–C_30_ were analysed under the same conditions to obtain linear retention index (LRI) values for the components.

### 2.6 Non-volatile compounds

#### 2.6.1 Sample preparation

An aliquot (1.5 ml) of melon juice was centrifuged at 7200*g* for 15 min and then the supernatant (400 μl) was transferred to an Amicon Ultra – 3000 MWCO filter unit (Millipore, Carrigtwohill, Co. Cork, Ireland) and centrifuged at 7200*g* for 30 min.

#### 2.6.2 Determination of free amino acids by GC–MS

An aliquot of the filtrate (100 μl) was derivatised using the EZ-Faast amino acid derivatisation technique (Phenomenex). GC–MS analysis of the derivatised samples was carried out using an Agilent 6890/5975 GC–MS instrument, as described by Elmore, Koutsidis, Dodson, Mottram, and Wedzicha (2005).

#### 2.6.3 Determination of organic acids and carbohydrates by capillary electrophoresis (CE)

An aliquot of the filtrate (100 μl) was analysed as described by Lignou et al. (2013).

### 2.7 Sensory analysis

The permanent in-house panel of 13 experienced assessors was used to develop a sensory profile to describe the sensory characteristics of the melon juice and the characteristics were estimated quantitatively. The same three replicates used for chemical analysis were also used for sensory analysis. Aliquots (20 ml) of melon juice (prepared as described above and filtered through a tea strainer to remove particulate matter) were presented to each assessor at room temperature in clear polypropylene tasting cups. During the development of the sensory profile, the assessors were asked to sniff and then taste (and swallow) the samples to produce as many descriptive terms as seemed appropriate. Reference materials (including a number of fruit and vegetables, such as strawberries, pineapple, aged apple and banana, citrus, plum, kiwi, butternut squash, different types of melon (honeydew and Galia), stored cantaloupe melon, pips and centre from cantaloupe melon, cucumber and other materials like sugar syrup) were used in order to help the assessors to standardise the language development process. These terms were discussed by the assessors, as a group, with the help of the panel leader, and this led to an agreed profile comprising 13 odour terms, 19 taste/flavour terms, 6 mouthfeel terms, and 10 after-effects terms. The quantitative sensory assessment took place in the sensory booths, each equipped with computer screen and a mouse. Compusense version 5 software (Compusense Inc., Guelph, Ontario, Canada) was used to acquire the sensory data. A warm-up sample (a mixture of the examined samples) was presented first, to eliminate first position bias and then the samples were presented to the assessors in a balanced randomised order. The assessors were instructed to sniff the samples to score the aroma attributes, and then taste (and swallow) the samples to score the overall taste/flavour attributes and the mouthfeel attributes. There was a 45-s pause after the end of the mouthfeel attributes and the assessors then scored the after-effects which included both taste and mouthfeel effects. The intensity of each attribute for each sample was recorded by the assessors on a 100-point unstructured line scale. Between samples, panellists cleansed their palate with yoghurt, cracker and water.

### 2.8 Statistical analysis

The quantitative data for each compound identified in the GC–MS analyses (volatile, semi-volatile and non-volatile compounds) were analysed by both one- and two-way analysis of variance (ANOVA) and principal component analysis (PCA) using XLSTAT Version 2012.1.01 (Addinsoft, Paris, France). For those compounds exhibiting significant difference in the one-way ANOVA, Fisher’s least significant difference (LSD) test was applied to determine which sample means differed significantly (*p* *<* 0.05). These data are shown in Table 1. SENPAQ version 3.2 (Qi Statistics, Reading, UK) was used to carry out ANOVA and PCA of sensory panel data. The means for the sensory data were taken over assessors and correlated with the means from instrumental data *via* multiple factor analysis (MFA) using XLSTAT.

## 3 Results

### 3.1 Volatile compounds

More than 70 compounds were identified in the headspace of the two genotypes. The most abundant compounds are listed in Table 1. These included 31 esters (acetates and non-acetate esters), 8 sulphur-containing compounds, 10 alcohols, 8 aldehydes, 2 terpene derivatives and 2 other compounds. Quantitative differences were observed between the two maturity stages (immature (i) and mature (m) fruit) and the two genotypes (medium shelf-life (MSL) and long shelf-life (LSL)). Esters (acetates and non-acetate esters) comprised more than 87% of the total volatiles collected from the iMSL fruit, a percentage which increased to more than 93% in the mMSL fruit. Similarly, the percentage of esters increased from 69% in the iLSL fruit to more than 77% in the mature fruit of the same genotype. The most abundant esters identified were ethyl acetate, 2-methylpropyl acetate, butyl acetate, 2-methylbutyl acetate and ethyl butanoate. Wyllie et al. (1996) and Bauchot et al. (2000) reported that these compounds were predominant in Makdimon (*C. melo* var. *reticulatus*) and Védrantais (*C. melo* var. *cantalupensis*) cultivars respectively. These compounds were also the most abundant in a number of Charentais cantaloupe cultivars (Aubert & Bourger, 2004) and in Jiashi muskmelon (var. *reticulatus*, Hami melon) (Pang, Guo, Qin, Yao, Hu, & Wu, 2012).

Both immature fruits contained very few esters compared to their respective mature fruit. Ten out of 13 acetates and 12 out of 18 non-acetate esters were found significantly higher in the mMSL fruit compared to the iMSL fruit. The same trend was observed for the LSL fruits, but the levels were much lower and the differences were not significant. However, the levels of ethyl esters and particularly ethyl acetate, ethyl propanoate, ethyl 2-methylpropanoate, ethyl butanoate and ethyl 2-methylbutanoate increased 4-fold for LSL and 26-fold for MSL with increasing maturity.

Generally, the levels of esters were remarkably lower in the LSL genotype, even in mLSL. Similar results were reported by Lamikanra et al. (2003), where hybrids with long shelf-life and hybrids with extended shelf-life presented significantly lower contents of total volatile aromas than traditional shelf-life *C. melo* var. *reticulatus* cv. Mission melons. Aubert and Bourger (2004), who studied the volatile compounds of 15 Charentais melon cultivars, reported the same trends: a reduction in a range of 43–77% of total esters in LSL melons compared to MSL or wild melons. They reported that these differences were more obvious for compounds with low odour threshold values, such as ethyl 2-methylbutanoate (0.006 μg/kg), ethyl butanoate (1 μg/kg), ethyl hexanoate (1 μg/kg), butyl acetate (2 μg/kg) and hexyl acetate (2 μg/kg). Bauchot et al. (1998) also noted that in transformed Charentais melons with an ACC oxidase antisense gene, the total volatiles were 60–85% lower than that of the nontransformed hybrids. They observed that the reduction in volatiles in these melons was greater for ethyl esters than for acetates, and since ethyl esters have lower odour threshold values than acetates, the reduction of ethylene production in these melons, had the greatest effect on the most potent odorants (Bauchot et al., 2000).

Eight sulphur-containing compounds were identified in the headspace of the samples including six thioether esters. Wyllie and Leach (1992) reported that 2-(methylthio)ethyl acetate and 3-(methylthio)propyl acetate were the dominant sulphur compounds in all melon cultivars studied, as was the case in the Charentais melon under study, but only in mMSL fruit. Ethyl 2-(methylthio)acetate was another important compound and again present only in mMSL fruit. Generally, the sulphur-containing esters were not detected in the LSL fruit and only two were detected in the iMSL fruit. These compounds are very important in the overall aroma profile of melons, because many are potent odorants with low odour thresholds. A few authors have reported that trace amounts of these compounds have a major impact on the musky note of some melon aromas (Wyllie & Leach, 1992; Hayata, Sakamoto, Kozuka, Sakamoto, & Osajima, 2002; Jordan, Shaw, & Goodner, 2001; Wyllie & Leach, 1990; Wyllie, Leach, Wang, & Shewfelt, 1994; Hayata, Sakamoto, Maneerat, Li, Kozuka, & Sakamoto, 2003). Aubert and Bourger (2004) also reported a considerable reduction in the levels of these compounds in LSL cultivars, whereas the total levels of them in wild or MSL cultivars were up to 17 times higher than in LSL cultivars.

Besides esters and sulphur-containing compounds, some alcohols and aldehydes were identified in the samples. The levels of most alcohols increased with increasing maturity for both genotypes, and this increase was significantly higher, particularly for mMSL fruit. Regarding the aldehydes found, no significantly changes were observed between the different samples except for 2-methyl-2-butenal and (Z)-6-nonenal. 2-Methyl-2-butenal was significantly higher in mMSL fruit and (Z)-6-nonenal was significantly higher in iMSL fruit. Terpenes like limonene, eucalyptol and geranylacetone were also found, however, only eucalyptol was found significantly higher in mMSL fruit. Finally, 2-methylbutanenitrile and 3-methylbutanentrile were reported for the first time in melons. These compounds were found to be significantly higher in mMSL fruit.

To sum up, among all the volatiles identified, 30 compounds were significantly affected by the maturity and 34 by the genotype, supporting the hypothesis that both factors were very important. The two-way ANOVA showed a clear trend, with many of the compounds (mainly esters, sulphur-containing compounds and several alcohols) showing a significant interaction between the two variables. The combination of an MSL variety, and a fruit harvested at maturity, produced a far greater increase in these compounds than would have been predicted from a simple additive model. This synergy is reflected in the GC–O data.

GC–olfactometry analysis of the samples yielded a total of 18 odorants in the chromatogram, which are presented in Table 2. All but one of these compounds were identified in the GC–MS analysis, the exception being (Z)-4-heptenal which was recognised by its characteristic aroma and confirmed by comparison of its LRI with that of the authentic sample. Quantitative differences were observed between the two maturity stages and the two genotypes. It is clearly illustrated in Table 2 that esters were the most important contributors to the desirable sweet and fruity aroma of the fruit. In particular, seven esters, including ethyl propanoate, propyl acetate, ethyl 2-methylpropanoate, methyl 2-methylbutanoate, ethyl butanoate, ethyl 2-methylbutanoate and butyl propanoate, contributed to the fruity, pineapple-like and sweet aroma, particularly of mMSL. Four of these esters were only detected in mMSL, and the other three branched esters were also detected in the less mature and the LSL fruits, but tended to have higher scores for mMSL.

Schieberle, Ofner, and Grosch (1990) studied the potent odorants in muskmelons by aroma extraction dilution analysis (AEDA), and they reported that indeed the volatile esters were responsible for the fruity notes in the aroma of muskmelon and that methyl 2-methylbutanoate and ethyl 2-methylbutanoate were the most intense odorants in the ester fraction. Jordan et al. (2001) also found that these two esters contributed to a fruity, sweet and cantaloupe-like aroma. Pang et al. (2012) studied the odour-active compounds of Jiashi muskmelon using both detection frequency analysis (DFA) and odour activity values (OAV). They reported that ethyl 2-methylpropanoate, ethyl butanoate and ethyl 2-methylbutanoate were the esters with the greatest relative importance and were characterised as having fruity, sweet and cantaloupe-like odours. Hexanal, which imparts a fresh green note (Schieberle et al., 1990), and (*Z*)-3-hexen-1-ol, which imparts a herbal green note (Jordan et al., 2001), were detected in these samples and described as having green and grass notes, respectively. Eucalyptol, reported by Schieberle et al. (1990), was another important odorant detected only in mMSL samples. Kemp, Knavel, and Stoltz (1972), and Kemp, Knavel, Stoltz, and Lundin (1974) concluded that (*Z*)-6-nonenal and 3,6-nonadien-1-ol were two potent odorants contributing to muskmelon flavour. These two compounds were also identified in these samples, having a cucumber and green note, respectively. (Z)-6-Nonenal was scored consistently higher in the immature fruits, consistent with the greener notes of under-ripe fruit. These compounds were also reported by Pang et al. (2012) in Jiashi muskmelons and, along with 2,6-nonadienal and 2-nonenal, were the important contributors for green and cucumber-like aromas. Pang et al. (2012) also stated that although esters were superior in concentration (86%), their contribution rate (OAV percentages) to the aroma profile of Jiashi muskmelons was only 10%, whereas alcohols and aldehydes were just the opposite. The contents of aldehydes and alcohols were only 11 and 4% that of esters, respectively, but their contribution rates were 56% and 34% respectively.

Finally, of the eight sulphur compounds which were identified in the headspace of the melons, four were detected by the assessors. S-Methyl 2-methylbutanethioate had a sulphury odour, whereas dimethyl trisulfide imparted a pickled onions and cabbage odour. Ethyl 2-(methylthio)acetate and ethyl 3-(methylthio)propanoate were only identified in mMSL and had an earthy but slightly cucumber note and a cardboard but slightly green odour, respectively. Overall, comparing the odours between the two maturity stages and the two genotypes, it can be observed that mMSL fruit presented the highest intensities, which resulted in a more aromatic fruit compared to the others.

### 3.2 Semi-volatile compounds

More than 40 compounds were identified in melon SPE extracts and 29 of them were quantified and listed in Table 1. Semi-volatile compounds included 9 esters (acetates and diacetates), 5 sulphur-containing compounds and a few other compounds (alcohols, aldehydes, furans, acids).

2,3-Butanediol diacetate and its precursor 2,3-butanediol monoacetate were identified and found to be significantly higher in mMSL genotype. These compounds were also identified in Japanese melon (cv. Golden Crispy) (Wyllie & Leach, 1990). 2,3-Butanediol diacetate possesses two asymmetric carbons (*erythro* and *threo* forms and a *meso*-form diastereoisomer), thus producing two peaks on GC (Aubert & Pitrat, 2006). According to Wyllie and Leach, (1990), the most abundant peak would be the D and/or L isomer, whereas the other would be the meso isomer. 1,2-Propanediol and 1,2-ethanediol diacetate were also identified and found to be significantly higher in mMSL genotype.

Five sulphur-containing compounds were identified with this method, three of which had been previously found in the headspace of these melons. The additional compounds were 2-(methylthio)-1-ethanol and 3-(methylthio)-1-propanol and these were, again, significantly higher in mMSL genotype. The relative quantities of these compounds showed good agreement between the two analytical methods.

Other compounds identified were alcohols, including 1-hexanol, (Z)-3-hexen-1-ol, benzyl alcohol and phenylethanol, compounds that increased with increasing maturity. 5,6,7,7a-Tetrahydro-4,4,7a-trimethyl-2[4*H*]-benzofuranone (dihydroactinidiolide) is potentially an important compound since it imparts a fruity musky note and was found in higher concentrations in the mature fruits. 2-Ethyl-4-hydroxy-5-methyl-3[2*H*]-furanone (homofuraneol) and 4-hydroxy-5-methyl-3[2*H*]-furanone (norfuraneol) were also identified in larger amounts in mature fruits of both genotypes. Finally hexadecanoic acid and 9-hexadecenoic acid were present in the extracts and increased as well with increasing maturity.

To sum up, among all the semi-volatiles identified, 17 compounds were significantly affected by maturity and only 11 by genotype, suggesting that the maturity factor was more important for this set of results. There was, again, a clear trend defined by two-way ANOVA where the majority of esters and sulfur-containing compounds showed a strong interaction between the variables, and the synergy between the maturity at harvest and genotype was evident.

GC–olfactometry analysis of the SPE extracts yielded a total of 20 aromatic regions in the chromatogram, which were described with a range of terms, including cabbage, cheesy, vinegar, Brie, mushroom, soil, bread, onions, balsamic, cucumber, green, vegetable, cooked potato, floral, synthetic, rubbery, woody, smoky, strawberry, caramel, candyfloss, and rose petals. A number of these odours were detected in our previous study (Lignou et al., 2013); however, the identities of many of these compounds remain unknown. A number of compounds were positively identified including (Z)-3-hexen-1-ol with a very strong cut grass odour in mMSL genotype. 2,3-Butanediol diacetate had an earthy, soily odour, and was also described by Wyllie, Leach, Wang, and Shewfelt (1995) as having an earthy note. Among the sulphur compounds, ethyl 2-(methylthio)acetate had a slight green odour, 3-(methylthio)propyl acetate had a mushroom-like odour and 3-(methylthio)-1-propanol an onion-like odour, respectively. Homofuraneol and norfuraneol were responsible for the strawberry sweet, caramel-like note in the aroma.

**Fig. 1:**
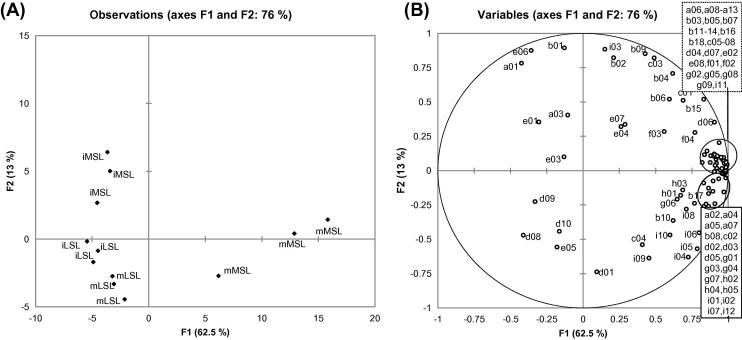
Principal component analysis of four different samples showing correlation with volatile and semi-volatile compounds. (A) Projection of the samples (MSL = medium shelf-life, LSL = long shelf-life, m = mature, i = immature); (B) Distribution of variables (codes on plot refer to compound codes in Table 1).

Principal component analysis was used to visualise graphically the differences in volatile and semi-volatile concentrations in the two maturity stages and the two genotypes. Twelve samples were used (2 maturity stages × 2 genotypes × 3 replicates) and 87 variables (61 volatile compounds and 26 semi-volatile compounds). The first two principal components accounted for 76% of the variation in the data (Fig. 1). The first axis mainly discriminated the mMSL fruit from the iMSL and the LSL genotype, whereas the second axis mainly discriminated the iMSL from the LSL genotype. For the LSL genotype, the immature and the mature fruits were not well separated on PC1 or PC2, and the effect of maturity at harvest for the LSL fruits was shown to be small compared to that for the MSL fruits. The distribution of the variables is shown in Fig. 1B. The majority of acetates (a02, a04-a13), non-acetate esters (b03, b05, b07, b08, b11-b14, b16, b18), diacetates (g02-g05, g08, g09), sulphur-containing compounds (c02, c05-c08 and h01-h05), several alcohols (d02-d05, d07, i01, i02, i07) and a few other compounds were positively correlated with the first axis. Methyl esters, including methyl acetate (a01), methyl propanoate (b01), methyl 2-methylpropanoate (b02), methyl butanoate (b04), methyl 2-methylbutanoate (b06), methyl pentanoate (b09) and methyl hexanote (b17), as well as S-methyl 2-methylbutanethioate (c03), (Z)-6-nonenal (e06) and 2,6-nonadienal (i03), were positively correlated with the second axis.

Mature MSL fruit, positively correlated with the first axis, was characterised by greater numbers of esters (including acetates, diacetates and non-acetate esters), sulphur-containing compounds, several alcohols and furans. Immature MSL, positively correlated with the second axis, was characterised by greater levels of methyl esters, (Z)-6-nonenal and 2,6-nonadienal. Immature LSL and mLSL fruit were negatively correlated with both first and second axis because the concentrations of esters (acetates, diacetates and non-acetate esters) were low and, moreover, sulphur-containing esters were not detected.

### 3.3 Non-volatile compounds

Two organic acids were identified: citric and malic acid (Table 1). Citric was the dominant acid in both maturity stages and genotypes. The levels of malic acid were approximately eight times lower than citric acid. The same acids were the dominant acids in cantaloupe melon (cv. Mission) (Lamikanra, Chen, Banks, & Hunter, 2000). Wang et al. (1996) found that citric acid increased slightly with increasing maturity in the melon of cv. Makdimon. This was also observed in our results; however, the increase of citric acid was not significant for either genotype (Table 1).

The sugars identified in the samples were glucose, fructose and sucrose. The results agree with those stated by Wang et al. (1996), Lester and Dunlap (1985), and Beaulieu, Lea, Eggleston, and Peralta-Inga (2003). As shown in Table 1, glucose and fructose decreased with increasing maturity, whereas sucrose increased significantly for both genotypes. Comparing the two genotypes, it can be seen that sucrose was significantly higher in LSL genotype. This probably happened because LSL fruit do not develop an abscission zone, and as a result the fruit may be harvested later, thus allowing for a longer period of sugar accumulation and higher sugar content (the major component of soluble solids in melon).

The dominant amino acids in both varieties (Table 1) were glutamine and aspartic acid; however, quantitative differences existed for a number of other amino acids between the maturity stages and genotypes. Almost all amino acids markedly increased with increasing maturity, except glutamine which decreased in the mMSL fruit, and leucine and isoleucine, which did not change significantly. Also alanine was found significantly higher in the mMSL fruit, whereas γ-ABA was one of the dominant amino acids in the LSL genotype.

It is well-known that there is a biogenetic relationship between the formation of certain aroma volatiles and levels of free amino acids (Wang et al., 1996). In particular, the amino acids alanine, valine, leucine, isoleucine and methionine are precursors of the majority of the esters found in melons (Bauchot et al., 1998; Wang et al., 1996; Wyllie et al., 1995). The trends observed in this study (increasing free amino acids during development and ripening, leucine and isoleucine remaining constant and glutamine decreasing) were also observed by Wang et al. (1996), who suggested that the type and extent of ester formation may be determined by substrate availability in the fruit. In mature melons, the total volatiles content is high, so considerable quantities of precursors are required for their formation. Although the concentrations of leucine and isoleucine remained constant during maturation, esters having carbon skeletons derived from isoleucine did increase with maturity. Wang et al. (1996) suggested that there is a series of steps in ester formation where a considerable degree of selectivity (enzymes involved) must happen as the substrates are drawn from the amino acid pool. Thus, the differences between cultivars in esters derived from amino acids are likely to be due to the efficiencies of the different enzyme pathways within each melon.

Consequently, it can be concluded that the extent of ester formation will depend on the amount of available substrates. Harvest time will influence the total volatile production, since fruit that was harvested prematurely would not accumulate sufficient concentrations of required volatiles substrates and this will lead to a poor flavour profile of that fruit. However, in addition to the availability of different substrates, subcellular localisation should be taken into account as well as the expression of synthesising enzymes, which play an important role in the reactions. Finally, the response to the climacteric genotypes (climacteric or non-climacteric) is also an important factor, since it was observed that the expression levels of genes responsible for biosynthesis of melon aroma volatiles are generally higher in climacteric genotypes as compared with non-climacteric genotypes (Gonda et al., 2010).

### 3.4 Sensory analysis

The sensory profile of the samples was generated by a trained panel of experts who, at the end of the profile development, agreed to use 49 terms for the quantitative assessment of the samples. Table 3 gives the mean panel scores for these attributes and significant differences for the samples, the assessors and their interactions as determined by ANOVA. This table shows 30 out of 49 attributes were found to be significantly different (3 nearly significantly different) between the four samples. A highly significant effect of assessor for all attributes was also found. This suggested that the assessors were using the scales differently; however, only a few attributes (mainly after-effects attributes) had a significant assessor × sample interaction, thus indicating that the assessors were ranking the samples in a similar way.

As shown in Table 3, sweet aroma, floral aroma and honey aroma were found to be significantly higher in mMSL, hence confirming the GC–MS results, where the levels of esters (acetates and non-acetate esters) were higher in these samples. These attributes were highly positively correlated with the sum of acetate and non-acetate esters, having correlation coefficients of more than 0.8 (data not shown). Brown orchard fruit aroma was also significantly higher in mMSL fruit. On the contrary, green and cucumber odour and taste/flavour attributes were scored significantly higher in iMSL fruit followed by iLSL fruit. This is also confirmed by both the GC-O and the GC–MS results which showed (Z)-6-nonenal (cucumber) was significantly higher in the immature fruit of both genotypes. Sweet and syrupy taste/flavour, as well as sweet aftertaste, were significantly higher in both maturity stages of LSL genotype and in mMSL fruit. This also agrees with the results for sucrose (Table 1).

**Fig. 2:**
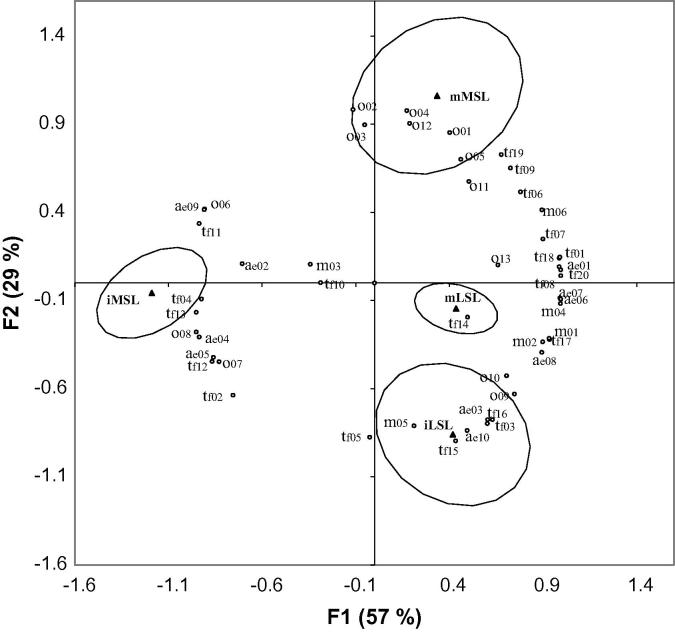
Principal component analysis of four different samples (▴) (MSL = medium shelf-life, LSL = long shelf-life, m = mature, i = immature) showing correlations with sensory attributes (○) (codes on plot refer to sensory attribute codes in Table 3).

Principal component analysis was carried out on the correlation matrix of all samples and all attributes (Fig. 2). The difference in maturity stage was the predominant distinguishing factor in the sensory analysis, with principal component 1 separating the immature from mature MSL fruit and principal component 2 separating the immature from the mature LSL and MSL fruits. Desirable sweet (o01), floral (o02), honey (o03), strawberries (o04) and ripe tropical fruit (o12) odour attributes, as well as floral (tf06), honey (tf07), strawberries (tf09) and ripe tropical fruit (tf19) taste/flavour attributes were associated with the mMSL fruit. On the other hand, cucumber odour (o07), cucumber taste/flavour (tf12), green odour (o08), green taste/flavour (tf13), acidic taste (tf04) and aftertaste (ae04), and savoury taste/flavour (tf02) were highly correlated with the iMSL fruit. Regarding the LSL genotype, earthy (o09-tf16) and musty (o10-tf17) odour and taste/flavour, and salty (tf03) taste/flavour attributes were associated with the iLSL fruit, whereas taste/flavour attributes like sweet (tf01), syrupy (tf08), brown orchard fruit (tf18), as well as sweet (ae01) aftertaste, were associated with the mLSL fruit. Similar results were reported by Beaulieu et al. (2004) who studied the effect of harvest maturity on the sensory characteristics of fresh-cut cantaloupe. They found that the maturity level at harvest coincided with significant differences in flavour attributes. Sweet aromatic flavour and taste significantly increased with increasing maturity, whereas cucurbit flavour decreased.

### 3.5 Multiple factor analysis (MFA)

**Fig. 3:**
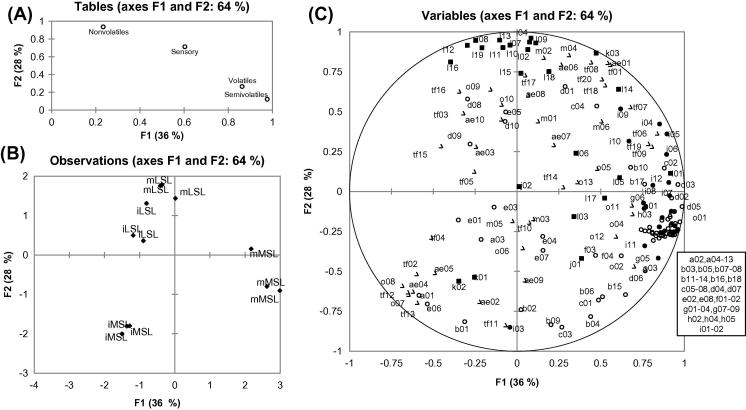
MFA: (A) Representation of groups (tables) of variables; (B) Representation of the samples (MSL = medium shelf-life, LSL = long shelf-life, m = mature, i = immature); (C) Distribution of variables (○ = volatiles, ● = semi-volatiles, ■ **= **non-volatiles and △ **= **sensory variables - codes on plot refer to codes in Tables 1 and 3).

MFA was used in order to simultaneously analyse several tables of variables (three tables for instrumental data: volatiles, semi-volatiles and non-volatiles and one table for sensory data), thus facilitating a study of the relationship between the observations (different samples), the variables and the tables. This was achieved by successively examining the PCA for each table, and then the value of the first eigen value of each analysis was used to weight the various tables in a further PCA. Finally, a weighted PCA on the columns of all the tables was performed (Pages, 2004). The coordinates of the tables were displayed and used to create the map of the tables (Fig. 3A). As it can be seen on the map, the first factor was related with the tables of volatiles, semi-volatiles and sensory attributes, whereas the second factor was mostly related with the non-volatiles but also with sensory tables.

The correlation maps of observations and variables are shown in Fig. 3B and C respectively. Although the plots do not implicitly detail coefficients of correlation, one can ascribe relative relationships between parameters closely related, and inversely related (separation close to 180°). Observing the variables map it can be concluded that the sensory analysis linked well with the instrumental data.

Mature MSL fruit was positively correlated with the first factor, in other words with sweet (o01), honey (o02), floral (o03) and strawberry (o04) odours and floral (tf06), honey (tf07), strawberries (tf09) and ripe tropical fruit (tf19) taste/flavour terms. These variables were then highly positively correlated with the majority of the esters, which are associated with desirable flavour. On the opposite side (negatively correlated with factor one and factor two), iMSL fruit was correlated with all the cucumber and green notes (o07, o08, tf12, tf13), as well as with acidic after-taste (ae04).

Compounds like (Z)-6-nonenal (e06) and two methyl esters (a01 and b01) were positively correlated with iMSL. It is interesting that 2,6-nonadienal (i03) was positively correlated with citrus taste/flavour (tf11). Additionally, the fact that this fruit was negatively correlated with sweet taste/flavour and after-effects terms, gave a fruit with an undesirable odour and taste. This can be drawn from the variables map, where all the esters are negatively correlated with iMSL fruit. Regarding the iLSL fruit (positively correlated with factor two), although it exhibited very low levels of esters compared to iMSL, the high concentration of sucrose and several amino acids contributing to taste (glutamic acid (l 1 6) and aspartic acid (l 1 2)), gave a fruit with an acceptable taste but lacking in desirable aroma. This was emphasised by high scores for earthy and musty odour, taste/flavour and after-effects (o09, o10, tf16, tf17, ae08).

Finally, mLSL was correlated with sweet (tf01) and syrupy (tf08) taste/flavour and sweet (ae01) after-effects terms. These terms were associated with sucrose (k03) and, indeed, this mLSL fruit contained the greatest quantity of sucrose. The slightly increased levels of esters (compared to iLSL and iMSL) gave a fruit with quite a nice odour and a very sweet taste.

## 4 Conclusion

Both sensory and instrumental analysis of volatile, semi-volatile and non-volatile compounds have identified significant differences between four melon samples that can be attributed to either the maturity stage or the genotype. The mature fruit of MSL exhibited the highest amount of esters (acetates, diacetates and non-acetate esters), and those melons were generally described by the assessors as having desirable fruity and sweet odours. Moreover, the combination of quite high sucrose levels, along with other compounds, like homofuraneol and norfuraneol, resulted in a fruit with a very sweet taste, while exhibiting the highest levels of strawberry taste/flavour and the lowest levels of bitter and acidic taste. The immature fruit of the MSL exhibited green, cucumber notes typical of an under-ripe melon and lacked the fruity flavour of the mature MSL. Both LSL melons, harvested immature and mature, were relatively sweet, with a sweet syrupy flavour but lacking in the fruity character of the mature MSL, exhibiting instead an earthy, musty quality. Overall, the mature MSL fruit was full of flavour confirming the hypothesis that fruit from MSL genotypes harvested mature will develop a strong aromatic flavour, whereas fruit either harvested too early or from LSL genotypes will develop a less aromatic flavour.

## Figures and Tables

**Table 1:** Approximate quantities of volatile, semi-volatile and non-volatile compounds identified in the headspace, SPE extracts or melon juice respectively of two genotypes of Charentais melon harvested at two different maturity stages.

Code	Compound	LRI^a^	ID^b^	Approximate quantity^c^				LSD^d^	P^e^
				iLSL	mLSL	iMSL	mMSL		
Volatile analysis									
*Acetates*									
a01	methyl acetate	<600	A	68^a^	53^a^	193^b^	37^a^	65	∗∗
a02	ethyl acetate	616	A	118^a^	458^a^	196^a^	3314^b^	512	∗∗∗
a03	1-methylethyl acetate	656	A	29	36	44	32	29	ns
a04	propyl acetate	715	A	16^a^	99^a^	49^a^	497^b^	154	∗∗∗
a05	2-methylpropyl acetate	773	A	134^a^	412^a^	214^a^	1469^b^	736	∗
a06	butyl acetate	817	A	18^a^	186^a^	92^a^	1538^b^	690	∗∗
a07	3-methylbutyl acetate	878	A	0.6^a^	2.7^a^	1.7^a^	24^b^	5.4	∗∗∗
a08	2-methylbutyl acetate	880	A	16^a^	61^a^	102^a^	1227^b^	685	∗∗
a09	pentyl acetate	915	A	nd	3.6^a^	3.4^a^	105^b^	59	∗∗
a10	(Z)-3-hexen-1-yl acetate	1005	A	34^a^	13^a^	46^a^	577^b^	380	∗
a11	hexyl acetate	1013	A	6.4^a^	36^a^	26^a^	598^b^	262	∗∗
a12	heptyl acetate	1111	A	nd	nd	nd	7.0		
a13	benzyl acetate	1168	A	1.3^b^	2.9^b^	nd	35^a^	28	ns^(0.060)^
*Non-acetate esters*									
b01	methyl propanoate	632	A	19^a^	16^a^	122^b^	38^a^	39	∗∗∗
b02	methyl 2-methylpropanoate	685	A	9.6^a^	12^a^	44^b^	29^a^^b^	25	∗
b03	ethyl propanoate	710	A	4.2^a^	24^a^	11^a^	559^b^	211	∗∗∗
b04	methyl butanoate	722	A	9.0^a^	8.0^a^	141^b^	159^b^	83	∗∗
b05	ethyl 2-methylpropanoate	758	A	nd	3.9^a^	1.5^a^	155^b^	60	∗∗∗
b06	methyl 2-methylbutanoate	782	A	21^a^	17^a^	98^b^	131^b^	54	∗∗
b07	ethyl butanoate	803	A	1.5^a^	15^a^	9.9^a^	1348^b^	590	∗∗
b08	propyl propanoate	814	A	nd	3.0^a^	nd	18^b^	11	∗
b09	methyl pentanoate	830	A	nd	nd	1.3	0.9	0.8	#
b10	isopropyl butanoate	844	A	0.4^a^	1.8^b^	0.8^a^	1.9^b^	0.8	∗∗
b11	ethyl 2-methylbutanoate	851	A	1.5^a^	7.6^a^	8.7^a^	422^b^	189	∗∗
b12	propyl butanoate	901	A	nd	nd	nd	30		
b13	ethyl pentanoate	903	A	nd	nd	nd	16		
b14	butyl propanoate	910	A	nd	0.9^a^	0.7^a^	4.0^b^	2.2	∗
b15	methyl hexanoate	926	A	nd	nd	4.3	7.9	5.0	#
b16	propyl 2-methylbutanoate	947	A	nd	0.1^a^	0.1^a^	2.3^b^	1.7	∗
b17	2-methylpropyl butanoate	956	A	nd	3.0^a^^b^	0.4^a^	4.5^b^	3.5	ns^(0.055)^
b18	ethyl hexanoate	999	A	nd	nd	nd	110		
*Sulphur-containing compounds*									
c01	*S*-methyl thioacetate	703	A	nd	nd	2.2	3.1	2.8	#
c02	dimethyl disulfide	748	A	3.4^a^	7.8^a^	2.0^a^	14^b^	6.0	∗∗
c03	*S*-methyl 2-methylbutanethioate	944	A	nd	nd	9.8	7.9	5.6	#
c04	dimethyl trisulfide	981	A	0.3	0.7	nd	0.5	0.5	#
c05	ethyl (methylthio)acetate	989	A	nd	nd	nd	52		
c06	2-(methylthio)ethyl acetate	1010	A	nd	nd	nd	69		
c07	ethyl 3-(methylthio)propanoate	1104	A	nd	nd	nd	8.0		
c08	3-(methylthio)propyl acetate	1127	A	nd	nd	nd	38		
*Alcohols*									
d01	2-methylpropanol	633	A	18^a^	63^b^	7.0^a^	34^a^^b^	35	∗
d02	1-butanol	668	A	2.1^a^	11^a^	4.1^a^	33^b^	9.6	∗∗∗
d03	2-methyl-1-butanol	749	A	36^a^	125^b^	28^a^	295^c^	71	∗∗∗
d04	(Z)-3-hexen-1-ol	866	A	5.5^a^	2.3^a^	3.0^a^	52^b^	13	∗∗∗
d05	1-hexanol	874	A	4.1^a^^b^	20^b^	2.0^a^	93^c^	17	∗∗∗
d06	eucalyptol	1041	A	1.1^a^	0.6^a^	4.9^a^	14^b^	8.2	∗
d07	1-octanol	1072	A	3.5^a^	5.1^a^	3.3^a^	35^b^	22	∗
d08	3-nonen-1-ol	1157	B	34^a^^b^	53^a^	15^b^	3.8^b^	44	ns^(0.073)^
d09	3,6-nonadien-1-ol	1165	B	14	10	3.6	1.7	18	ns
d10	1-nonanol	1173	A	21	27	8.2	10	28	ns
*Aldehydes*									
e01	2-methylbutanal	666	A	4.8	6.0	8.0	3.4	8.0	ns
e02	2-methyl 2-butenal	745	A	0.5^a^	1.5^a^	0.7^a^	9.8^b^	4.5	∗∗
e03	hexanal	811	A	9.4	17	17	11	13	ns
e04	heptanal	907	A	8.0	7.6	9.0	9.0	6.7	ns
e05	benzaldehyde	974	A	9.9^a^^b^	31^b^	6.6^a^	6.5^a^	23	ns
e06	(Z)-6-nonenal	1104	A	2.0^a^	nd	13^b^	nd	5.4	∗
e07	nonanal	1108	A	30	27	36	35	33	ns
e08	decanal	1210	A	18^a^	16^a^	16^a^	36^b^	17	ns^(0.062)^
*Other compounds*									
f01	2-methylbutanenitrile	728	A	nd	0.4^a^	1.1^a^	56^b^	18	∗∗∗
f02	3-methylbutanenitrile	735	A	nd	nd	0.6^a^	18^b^	5.9	∗∗∗
f03	limonene	1036	A	1.3	1.7	1.9	2.4	1.4	ns
f04	geranylacetone	1451	A	nd	0.2	1.3	4.4	5.0	Ns
Semi-volatile analysis									
*Esters*									
g01	2-acetoxy-3-butanone	1358	A	nd	nd	nd	4.6		
g02	2,3-butanediol diacetate^f^	1462	A	0.1^a^	0.8^a^	0.6^a^	8.5^b^	4.6	∗∗
g03	1,2-propanediol diacetate	1486	A	nd	0.2^a^	0.1^a^	0.6^b^	0.3	∗
g04	2,3-butanediol diacetate^f^	1497	A	0.1^a^	0.8^a^	0.2^a^	6.1^b^	1.5	∗∗∗
g05	1,2-ethanediol diacetate	1518	A	0.1^a^	0.6^a^	0.9^a^	2.5^b^	1.0	∗∗
g06	2,3-butanediol monoacetate^g^	1536	A	0.2^a^	0.6^a^	0.2^a^	10^b^	6.5	∗
g07	2,3-butanediol monoacetate^g^	1549	A	0.2^a^	1.1^a^	0.8^a^	30^b^	4.3	∗∗∗
g08	1,3-butanediol diacetate	1593	B	nd	nd	nd	1.0		
g09	1,4-butanediol diacetate	1748	B	nd	nd	nd	1.2		
*Sulphur-containing compounds*									
h01	ethyl (methylthio)acetate	1423	A	nd	nd	nd	6.3		
h02	2-(methylthio)ethyl acetate	1468	A	nd	nd	nd	21		
h03	2-(methylthio)ethanol	1503	A	nd	nd	nd	4.6		
h04	3-(methylthio)propyl acetate	1601	A	nd	nd	nd	14		
h05	3-(methylthio)-1-propanol	1689	A	nd	nd	nd	5.2		
*Other*									
i01	1-hexanol	1336	A	0.3^a^	1.4^a^	0.2^a^	13^b^	2.8	∗∗∗
i02	(Z)-3-hexen-1-ol	1363	A	1.1^a^	0.3^a^	0.6^a^	14^b^	1.9	∗∗∗
i03	2,6-nonadienal	1557	A	0.2^a^^b^	0.1^a^	0.6^c^	0.4^b^^c^	0.2	∗∗
i04	benzyl alcohol	1844	A	8.7^b^	17^c^	1.5^a^	23^c^	5.7	∗∗∗
i05	phenylethanol	1879	A	1.2^b^	2.6^c^	0.2^a^	3.7^d^	0.8	∗∗∗
i06	dihydro-3-hydroxy-4,4-dimethyl-2(3H)-furanone	1995	B	0.3^a^	1.0^b^	0.1^a^	1.6^c^	0.4	∗∗∗
i07	benzenepropanol	2014	B	0.2^a^	0.6^a^	nd	3.2^b^	1.0	∗∗∗
i08	2-ethyl-4-hydroxy-5-methyl-3(*2H*)-furanone	2064	A	nd	0.6^a^	nd	2.5^b^	1.5	∗
i09	4-hydroxy-5-methyl-3(*2H*)-furanone	2081	A	2.0^a^	15^b^	0.6^a^	13^b^	6.2	∗∗
i10	5,6,7,7a-tetrahydro-4,4,7a-trimethyl-2(4H)-benzofuranone	2315	A	0.5^a^	2.3^b^	0.8^a^	2.1^b^	0.5	∗∗∗
i11	hexadecanoic acid	2886	A	14^a^	34^a^	33^a^	56^b^	22	∗
i12	9-hexadecenoic acid	2928	B	5.9^a^^b^	17^b^	4.3^a^	31^c^	13	∗∗
Non-volatile analysis									
*Organic acids*									
j01	citric acid			3.1	3.4	4.0	4.5	1.5	ns
j02	malic acid			0.4	0.5	0.5	0.4	0.2	ns
*Sugars*									
k01	fructose			14	13	20	14	9.4	ns
k02	glucose			13	10	19	11	9.1	ns
k03	sucrose			57^b^	84^c^	15^a^	67^b^	16	∗∗∗
*Free amino acids*									
l01	Ala			299^a^	714^b^	271^a^	1384^c^	361	∗∗∗
l02	Gly			103^b^	228^c^	37^a^	92^b^	37	∗∗∗
i03	a-ABA			6.0^a^	9.0^a^^b^	9.0^a^^b^	10^b^	3.0	∗
l04	Val			216^b^	348^c^	59^a^	169^b^	69	∗∗∗
l05	Leu			25	31	25	39	17	ns
l06	Ile			40	37	33	42	13	ns
l07	Thr			121^b^	174^c^	63^a^	109^a^^b^	46	∗∗
l08	γ-ABA			1485^b^	2216^c^	371^a^	515^a^	388	∗∗∗
l09	Ser			402^b^	623^c^	162^a^	336^a^^b^	193	∗∗
l10	Pro			65^c^	99^d^	26^a^	44^b^	13	∗∗∗
l11	Asn			171^b^	252^c^	111^a^	136^a^^b^	43	∗∗∗
l12	Asp			3544^b^	5627^c^	1294^a^	1243^a^	1015	∗∗∗
l13	Met			63^c^	106^d^	21^a^	37^b^	12	∗∗∗
l14	Glu			305^a^^b^	568^b^	15^a^	589^b^	363	∗
l15	Phe			62^b^	129^c^	27^a^	49^a^^b^	49	∗∗
l16	Gln			6449^b^	8659^b^	3176^a^	2460^a^	2515	∗∗
l17	Lys			19	21	20	28	13	ns
l18	Tyr			21^a^	31^b^	14^a^	22^a^^b^	9.0	∗
l19	Trp			21^b^	33^c^	7.0^a^	10^a^	6.0	∗∗∗

a For compounds a to f: linear retention index on DB-5 column, for compounds g to i: linear retention index on a DB-WAX.

b A, mass spectrum and LRI agree with those of authentic compound; B, mass spectrum agrees with reference spectrum in the NIST/EPA/NIH mass spectra database and LRI agree with those in the literature.

c For compounds a to f: estimated quantities (ng) collected from the headspace of 2 ml of melon juice diluted in 10 ml of HPLC water, calculated by comparison with 130.6 ng of 1,2-dichlorobenzene used as internal standard; for compounds g to i: estimated quantities (mg) from 20 ml melon juice, calculated by comparison with 100 mg of 3-chlorophenol used as internal standard; for compounds j and k: estimated quantities (g/l) in melon juice and for compounds l: estimated quantities (mg/l) in melon juice; means not labelled with the same letters are significantly different (*p* < 0.05); means of three replicate samples; nd, not detected.

d Least significant difference at *p* = 0.05.

e Probability, obtained by ANOVA, that there is a difference between means; ns, no significant difference between means (*p* > 0.05); ⁎ significant at the 5% level; ⁎⁎ significant at the 1% level; ⁎⁎⁎ significant at 0.1% level, # difference between samples (absent *vs*. present) but no significant difference between those samples where the compound was present.

fg Pair of diastereoisomers.

**Table 2:** Odorants identified by GC-O/MS in the headspace of two genotypes of Charentais melon harvested at two different maturity stages.

Code	Compound	LRIexpt^a^	Odour description	Intensity^b^			
				iLSL	mLSL	iMSL	mMSL
1	ethyl propanoate	713	fruity, over-ripe	-	-	-	9
2	propyl acetate	715	pungent, sweet fruit	-	-	-	12
3	ethyl 2-methylpropanoate	759	fruity, pineapple	-	10	6	12
4	methyl 2-methylbutanoate	778	fruity, pineapple	9	11	9	11
5	hexanal	805	green, grass	4	9	7	6
6	ethyl butanoate	806	sweet fruity, fake sweets	-	-	-	10
7	ethyl 2-methylbutanoate	849	fruity sweet, pineapple	8	11	8	13
8	(Z)-3-hexen-1-ol	856	fresh-cut grass	-	-	-	5
9	1-hexanol	870	herbaceous	-	-	-	5
10	(Z)-4-heptenal	902	lamb fat, cheesy	-	-	11	-
11	butyl propanoate	911	ripe banana	-	-	-	4
12	*S*-methyl 2-methylbutanethioate	940	sulphury	-	-	5	3
13	dimethyl trisulfide	972	pickled onions, cabbage	10	13	9	13
14	ethyl (methylthio)acetate	985	earthy, slightly cucumber	-	-	-	5
15	eucalyptol	1032	pine	-	-	-	3
16	ethyl 3-(methylthio)propanoate	1102	cardboard, slightly green	-	-	-	4
17	(Z)-6-nonenal	1110	cucumber	10	-	12	-
18	3,6-nonadien-1-ol	1164	rags, dry	8	5	4	3

a Linear retention index on DB-5 column, calculated from a linear equation between each pair of straight chain *n*-alkanes C_6_-C_25_.

b The sum of intensities recorded by two assessors for each sample (scoring scale: weak = 3, medium = 5, strong = 7), − = not detected.

**Table 3:** Mean panel scores for sensory attributes of two genotypes of Charentais melon harvested at two different maturity stages.

Code	Attribute	Score^a^				LSD^b^	P^c^		
		iLSL	mLSL	iMSL	mMSL		S	A	I
Odour									
o01	sweet	41^b^	41^b^	40^b^	50^a^	5.4	∗∗	∗∗∗	ns
o02	floral	17^b^	19^b^	21^a^^b^	26^b^	6.4	∗	∗∗∗	ns
o03	honey	11^b^	10^b^	14^b^	21^a^	4.4	∗∗∗	∗∗∗	ns
o04	strawberries	6.5^b^	10^a^^b^	8.8^b^	14^a^	4.4	∗∗	∗∗∗	ns
o05	orange squash	13^a^	18^a^	14^a^^b^	18^a^	4.4	ns	∗∗∗	ns
o06	citrus	10	10	11	10	2.8	ns	∗∗∗	ns
o07	cucumber	17^b^	12^c^	22^a^	12^c^	4.1	∗∗∗	∗∗∗	ns
o08	green^d^	14^b^	14^b^	21^a^	11^b^	4.0	∗∗∗	∗∗∗	ns
o09	earthy	18^a^	14^a^^b^	8.1^c^	11^b^^c^	5.5	∗∗	∗∗∗	ns
o10	musty	16^a^	8.9^b^	5.1^b^	9.0^b^	6.3	∗∗	∗∗	ns
o11	brown orchard fruit^e^	13^a^^b^	10^b^	9.9^b^	17^a^	4.4	∗∗	∗∗∗	ns
o12	ripe tropical fruit^f^	11	11	11	14	3.8	ns	∗∗∗	ns
o13	fermenting	13^a^	9.9^b^	9.2^b^	13^a^	2.9	∗∗	∗∗∗	ns
Taste/Flavour									
tf01	sweet	60^a^	66^a^	31^b^	65^a^	8.8	∗∗∗	∗∗∗	∗∗
tf02	savoury	15^a^^b^	12^b^^c^	17^a^	11^c^	3.1	∗∗∗	∗∗∗	ns
tf03	salty	18^a^	15^a^^b^	13^b^	13^b^	4.3	ns	∗∗∗	ns
tf04	acidic	15	17	20	15	4.7	ns	∗∗∗	ns
tf05	bitter	17	14	15	13	4.8	ns	∗∗∗	ns
tf06	floral	21^a^	19^a^^b^	14^b^	26^a^	6.3	∗∗	∗∗∗	∗
tf07	honey	17^a^	14^a^	9.2^b^	18^a^	5.1	∗∗	∗∗∗	ns
tf08	syrupy	37^a^	41^a^	10^b^	37^a^	9.4	∗∗∗	∗∗∗	∗∗
tf09	strawberries	7.5^b^	7.9^b^	3.5^b^	13^a^	4.8	∗∗	∗∗∗	∗
tf10	orange squash	11	9.1	11	11	4.7	ns	∗∗∗	ns
tf11	citrus	6.4^b^	6.5^b^	11^a^	8.4^b^	2.7	∗∗	∗∗∗	ns
tf12	cucumber	16^b^	10^b^^c^	23^a^	9.4^c^	6.4	∗∗∗	∗∗∗	∗∗∗
tf13	green	11^b^	8.5^b^	17^a^	9.8^b^	4.1	∗∗∗	∗∗∗	ns
tf14	metallic	22^a^	17^b^	17^a^	20^a^^b^	3.9	ns^(0.050)^	∗∗∗	ns
tf15	pithy	17	16	13	12	7.6	ns	∗∗	∗∗
tf16	earthy	22^a^	17^b^	11^b^	11^b^	5.7	∗∗	∗∗∗	ns
tf17	musty	18^a^	15^a^	5.4^b^	13^a^	6.1	∗∗∗	∗∗∗	ns
tf18	brown orchard fruit^e^	17^a^	17^a^	6.9^b^	18^a^	6.3	∗∗	∗∗	ns
tf19	ripe tropical fruit^f^	9.8^b^	13^a^^b^	8.1^b^	16^a^	5.7	∗	∗∗∗	∗
tf20	fermenting	15^a^	15^a^	4.9^b^	16^a^	7.5	∗∗	∗∗∗	∗∗
Mouthfeel									
m01	mouth drying	41	41	37	40	6.2	ns	∗∗∗	ns
m02	mouth coating	41^a^^b^	43^a^	32^c^	37^b^^c^	5.5	∗∗	∗∗∗	ns
m03	tongue tingling	8.0	6.9	7.9	7.9	4.0	ns	∗∗∗	ns
m04	body	46^a^	46^a^	24^b^	42^a^	7.5	∗∗∗	∗∗∗	∗
m05	salivating	33	32	32	32	5.7	ns	∗∗∗	ns
m06	smoothness	44^a^^b^	44^a^^b^	37^b^	47^a^	6.5	ns^(0.052)^	∗∗∗	ns
After-effects									
ae01	sweet	50^a^	55^a^	26^b^	52^a^	10	∗∗∗	∗∗	∗∗∗
ae02	savoury	14	11	16	14	4.8	ns	∗∗∗	∗
ae03	salty	15	15	13	13	4.4	ns	∗∗∗	∗
ae04	acidic	15^a^^b^	13^b^	21^a^	13^b^	5.7	∗	∗∗∗	∗
ae05	bitter	16^a^^b^	14^b^	19^a^	14^b^	4.2	ns ^(0.050)^	∗∗∗	ns
ae06	mouthcoating	42^a^	43^a^	33^b^	41^a^	4.6	∗∗	∗∗∗	∗
ae07	drying	42	43	39	42	7.8	ns	∗∗∗	∗∗∗
ae08	musty	21^a^	17^a^	8.4^b^	15^a^^b^	7.3	∗	∗∗∗	∗
ae09	soapy	4.5	5.2	8.9	6.6	5.0	ns	∗∗∗	ns
ae10	metallic	22	22	19	18	6.5	ns	∗∗∗	∗∗

a Means not labelled with the same letters are significantly different (*p* < 0.05); means are from three replicate samples.

b Least significance difference at *p* = 0.05.

c Probability, obtained from ANOVA, that there is a difference between means; ns, no significant difference between means (*p* > 0.05); ⁎ significant at the 5% level; ⁎⁎ significant at the 1% level; ⁎⁎⁎ significant at the 0.1% level; *F*-ratios for sample and assessor were calculated by comparing the mean square of the effect with the mean square of the sample×assessor interaction; S: significance of samples, A: significance of assessors, I: significance of the interaction (S×A).

d Odour associated with freshly cut grass and green beans.

e Odour or taste-flavour associated with overripe apples and pears.

f Odour or taste-flavour associated with ripe bananas and pineapples.
